# Fgf negative regulators control early chick somite myogenesis

**DOI:** 10.1186/1471-2164-15-S2-P18

**Published:** 2014-04-02

**Authors:** Muhammad Abu-Elmagd, Katarzyna Goljanek-Whysall, Grant Wheeler, Andrea Münsterberg

**Affiliations:** 1 Centre of Excellence in Genomic Medicine Research, King Abdulaziz University, Jeddah, Kingdom of Saudi Arabia; 2 School of Biological Sciences, University of East Anglia, Norwich, UK; 3 Zoology Department, Faculty of Sciences, Minia University, Minia, Egypt; 4 Department of Musculoskeletal Biology, Institute of Ageing and Chronic Disease, Faculty of Health and Life Sciences, University of Liverpool, Liverpool, UK

## Background

Negative regulators of the signalling transduction cascades have been shown to play critical role in controlling different aspects of normal embryonic development [1,2]. It is believed that these regulators control FGF signalling through a negative feedback mechanism [3]. The role of the FGF negative regulators during somite myogenesis is still not clear. In the current study, we tried to shed some light on FGF signalling through their negative regulators during early chick somite myogenesis.

## Materials and methods

Chick embryos at HH7-HH25 were obtained by incubating white leghorn fertilised eggs at 38ºC for the desired times. Single and double *In situ* hybridisation for the FGF negative regulators with other myogenic markers at different stages of chick embryos were carried out. Embryos were then sectioned and their expression pattern was critically analysed. For the functional analysis, epithelial somites were targeted for injection of different expression constructs. Embryos were then fixed and whole mount *in situ* hybridisation for MyoD and Mgn probes was carried out.

## Results

Our results show that a number of FGF negative regulators are expressed in somites and their expression overlaps with that of MyoD and Mgn expression. Using gain– and loss-of-function approach, a number of the FGF negative regulators show that they can block FGF expression.

## Conclusions

A number of FGF negative regulators are expressed during early chick somites formation. These regulators are able to regulate FGF activation of somite myogenesis. Our study shed some light on FGF signalling and their negative regulators during early chick somite myogenesis.

The authors would like to thank KACST, Saudi Arabia for funding the research project (Project Code: 13-BIO789-03).
